# Timing of Favorable Conditions, Competition and Fertility Interact to Govern Recruitment of Invasive Chinese Tallow Tree in Stressful Environments

**DOI:** 10.1371/journal.pone.0071446

**Published:** 2013-08-13

**Authors:** Christopher A. Gabler, Evan Siemann

**Affiliations:** 1 Department of Ecology and Evolutionary Biology, Rice University, Houston, Texas, United States of America; 2 Department of Biology and Biochemistry, University of Houston, Houston, Texas, United States of America; CNRS/Université Joseph-Fourier, France

## Abstract

The rate of new exotic recruitment following removal of adult invaders (reinvasion pressure) influences restoration outcomes and costs but is highly variable and poorly understood. We hypothesize that broad variation in average reinvasion pressure of *Triadica sebifera* (Chinese tallow tree, a major invader) arises from differences among habitats in spatiotemporal availability of realized recruitment windows. These windows are periods of variable duration long enough to permit establishment given local environmental conditions. We tested this hypothesis via a greenhouse mesocosm experiment that quantified how the duration of favorable moisture conditions prior to flood or drought stress (window duration), competition and nutrient availability influenced *Triadica* success in high stress environments. Window duration influenced pre-stress seedling abundance and size, growth during stress and final abundance; it interacted with other factors to affect final biomass and germination during stress. Stress type and competition impacted final size and biomass, plus germination, mortality and changes in size during stress. Final abundance also depended on competition and the interaction of window duration, stress type and competition. Fertilization interacted with competition and stress to influence biomass and changes in height, respectively, but did not affect *Triadica* abundance. Overall, longer window durations promoted *Triadica* establishment, competition and drought (relative to flood) suppressed establishment, and fertilization had weak effects. Interactions among factors frequently produced different effects in specific contexts. Results support our ‘outgrow the stress’ hypothesis and show that temporal availability of abiotic windows and factors that influence growth rates govern *Triadica* recruitment in stressful environments. These findings suggest that native seed addition can effectively suppress superior competitors in stressful environments. We also describe environmental scenarios where specific management methods may be more or less effective. Our results enable better niche-based estimates of local reinvasion pressure, which can improve restoration efficacy and efficiency by informing site selection and optimal management.

## Introduction

Reinvasion pressure, or the rate of new exotic recruitment following removal of mature conspecifics, varies broadly among similarly invaded habitats and is crucial to restoration outcomes and costs but is poorly understood and difficult to predict [1]–[3]. When restoring habitats dominated by an exotic plant, invader density governs strength of impacts on communities and ecosystem functions [4] and influences required management methods, which can have diverse impacts on non-target species [5] and vary widely in cost [6]. Accurately estimating reinvasion pressure can improve restoration efficacy and efficiency by informing site selection and optimal management strategies [2], but the mechanisms driving its variation are poorly understood despite their importance to restoration and exotic plant control [3].

*Triadica sebifera* (Chinese tallow tree) is a major invader in the southeastern United States that exhibits broad variation in average reinvasion pressure during restorations of habitats it previously dominated [7] (Gabler & Siemann, unpublished data). We hypothesize that this variation arises predominantly because differences among invaded habitats in their temporal availability of moisture conditions suitable for *Triadica* recruitment drive differences in average *Triadica* recruitment success [2]. Differences in average reinvasion pressure can become masked over time by *Triadica* dominance because ontogenetic niche expansions (increases in niche breadth during development) enable *Triadica* to persist in moisture conditions unsuitable for its recruitment [8].

Our ‘outgrow the stress’ hypothesis further posits that short-term reinvasion pressure depends on propagule abundance and spatiotemporal availability of realized recruitment windows, which are akin to ‘safe sites’ [9] but emphasize ontogenetic niche expansions [2]. Realized recruitment windows are periods of variable duration that permit exotics with expanding niches to become established and are determined by abiotic conditions and interspecific interactions with recipient communities. This hypothesis stresses factors that influence seedling growth during temporary periods of suitable environmental conditions and may thus influence establishment success, which Holmgren *et al.* [10] demonstrate can influence vegetation structure on a landscape scale and are central to the present work.

Existing hypotheses explaining recruitment or invasion success have long emphasized spatial and/or temporal availability of conditions suitable for germination and establishment. Harper [9] defined ‘safe sites’ as sites free of specific hazards, e.g. intolerable moisture conditions, and argued that all colonization occurs as a function of their availability. Grubb [11] described similar ‘regeneration niches’ and Johnstone [12] elaborated safe sites to consider their dispersion in time. These suggest strict limits in resource availability or climatic tolerances define recruitment opportunities, and indeed many studies demonstrate that temporary variation across distinct abiotic thresholds can permit or preclude plant establishment in stressful habitats [13], [14].

More recent spatiotemporal niche-based invasion hypotheses consider a broader range of biotic and abiotic factors and place greater emphasis on their interactions, e.g. competition with resident species for fluctuating resources [15]. Such hypotheses recognize the importance of stochastic disruptions to communities, like earlier theories, but accentuate historical contingencies arising from these and other irregular events, e.g. ‘niche opportunity’ of Shea and Chesson [16]. They also identify “grayer” abiotic thresholds resulting from stress mediating effects of certain biotic interactions [17] or life history strategies, e.g. the storage effect [18]. Our ‘outgrow the stress’ hypothesis takes spatiotemporal niche-based invasion hypotheses one step further – albeit strictly in the context of reinvasion – by considering not only fluctuations in the biotic and abiotic environment but also ontogenetic changes in invader environmental tolerances and ultimately the impacts of environment on ontogenetic development of invaders and thus their individual tolerances through time [2].

Availability of soil resources, including water, and competition for these and other resources are fundamental factors limiting plant distributions [19], [20], so we expect they are principally important in defining realized recruitment windows. Nutrient availability has been show to have strong effects on invasion success [21]–[23]. Fertilization increased *Triadica* invasion pressure in coastal prairies by increasing seedling survival, height and biomass [24], but increased *Triadica* survival in coastal prairies with nutrient addition was not always observed and performance benefits were nutrient specific [25].

Water regime is crucial to *Triadica*’s local distribution and can vary considerably on small spatial scales in its introduced range [26]. Though established *Triadica* seedlings have relatively broad moisture tolerances [27], [28], moisture requirements for germination and survival and growth of young seedlings are relatively narrow [8]. In other systems, interannual variation in precipitation can influence establishment success among years [29] and cause episodic recruitment [30]. Preliminary results from experimental restorations of eleven sites dominated by *Triadica* suggest reinvasion pressure correlates with soil moisture, and that addition of native seeds may decrease *Triadica* recruitment success in favorable moisture conditions but, at least early in restoration, increase recruitment success in more stressful conditions (Gabler & Siemann, unpublished data). Interactions between these fundamental factors remain unclear, especially in high stress environments where they may be most important to *Triadica* recruitment success.

We began validating our ‘outgrow the stress’ hypothesis by demonstrating that *Triadica* undergoes rapid ontogenetic moisture niche expansions, which enable seedlings to tolerate conditions that do not permit germination (i.e. continuous flooding and short-term drought) within two months of germination [8]. In this work, we continue vetting this hypothesis by investigating realized recruitment windows. Here we quantify how the duration of favorable moisture conditions prior to flood or drought stress (window duration), competition and nutrient availability influence *Triadica* recruitment within highly stressful but variable environments.

Greater understanding of how temporal moisture fluctuations, competition and nutrient availability influence *Triadica* survival and performance would improve estimates of average reinvasion pressure within particular habitats, and would enhance our ability to predict short-term reinvasion pressure based on climate forecasts [2], [31]. Knowledge of context-dependent effects of native seed addition or fertilization on *Triadica* recruitment would promote management plans better fit to local circumstances and more able to mitigate or exploit stochastic events such as extreme weather or nutrient or seed pulses. Both provide valuable decision-making tools for restoration and *Triadica* management, and these approaches are applicable in other invaded systems.

We investigated how window duration and key ecological factors influence *Triadica* recruitment in stressful environments by performing a mesocosm experiment manipulating window duration, stress type, competition and fertilization. If availability of realized recruitment windows governs recruitment in stressful habitats, longer window durations prior to stress should increase *Triadica* abundance and performance once stress resumes. If size confers tolerance in plants with ontogenetic niche expansions [32], factors influencing growth rates should affect recruitment during finite windows of opportunity [2], thus fertilization and competition should increase and decrease *Triadica* success, respectively. To better understand how temporal availability of realized recruitment windows influence exotic recruitment and key biotic and abiotic factors shape realized recruitment windows, we ask: (i) How do window duration, competition and fertilization interact to influence *Triadica* seedling abundance and performance? (ii) How does the nature of water stress influence *Triadica* success and/or alter the effects of other factors?

## Methods

### Focal Species

Chinese tallow tree [*Triadica sebifera* (L.) Small, Euphorbiaceae; synonym *Sapium sebiferum;* ‘*Triadica*’ throughout] is a major invasive species in the southeastern United States naturalized from Texas to Arkansas and eastward from Florida to North Carolina and in California [26], [33]. *Triadica* aggressively displaces native plants in grasslands (e.g. imperiled coastal prairies), wetlands and forests and can form monocultures in only two decades [26], [34]. *Triadica* is a superior competitor due to a combination of high growth rates [35], prolific seed production [36], broad abiotic tolerances [27], [37] and low herbivore loads in its introduced range [38].

*Triadica* seeds exhibit dormancy and can remain viable in seed banks for 5+ years [26]. Seeds require specific abiotic conditions to cue germination, namely widely oscillating day-night temperatures, which are characteristic of exposed soil and promote *Triadica* germination in disturbed conditions [7], [39], [40], and moist but unsaturated soils, which promote germination in moisture conditions optimal for seedling survival and growth [8]. Established *Triadica* juveniles have broad moisture tolerances [27], [28], but the moisture requirements of newly germinated *Triadica* seedlings are relatively narrow and rapidly broaden in the first months of development to enable persistence in conditions ranging from constant flooding to short-term drought (an ontogenetic moisture niche expansion); survival in flooded conditions depends strongly on plant size, specifically whether seedlings have any emergent leaves [8]. We expect rapid moisture niche expansions early in ontogeny are crucial to *Triadica* establishment success during brief windows of favorable conditions in temporally variable environments. Furthermore, we hypothesize that size confers moisture tolerance and thus factors such as competition and nutrient availability also influence minimum establishment time and recruitment success during abiotic windows of opportunity [2].

### Greenhouse Mesocosm Experiment

We manipulated duration of favorable moisture conditions prior to water stress (window duration), competition and nutrient availability in mesocosms and quantified *Triadica* abundance, survival and performance through a period of water stress. Our balanced full factorial design used 2.8 L pots with five window duration, two competition, two fertilization and two stress type treatments with 10 replicates per treatment combination (n = 400 pots). In July 2008 we filled 2.8 L tapered square plastic Treepots (36 cm tall, 6–10 cm diameter; Stuewe & Sons, Oregon, USA) with ∼2 L field soil collected from Justin Hurst Wildlife Management Area (JHWMA) in southeast Texas and randomly assigned treatments to each. Soils collected near 28.959502 N, −95.461348 W were expansive Pledger (85%) and Brazoria Clay (10%) vertisols (very-fine, smectitic, hyperthermic Typic Hapluderts) with 60–80% clay content. JHWMA is limited-access public land owned and managed by the Texas Parks & Wildlife Department, whose staff granted us access and permission to collect soil. We added 10 washed *Triadica* seeds to each pot from a well-mixed batch collected in 2007 from source trees in southeast Texas. This provided relatively dense seed banks (∼1000 seeds/m^2^) typical of habitats dominated by *Triadica*. We housed pots in a climate controlled greenhouse under natural light with day temperatures of 29–31°C and night temperatures of 19–21°C, which approximates spring in southeast Texas. See Gabler and Siemann [8] for additional site description and seed preparation protocols.

We established five window duration treatments by exposing pots to identical well-drained and well-watered conditions for 4, 6, 8, 10 or 12 weeks before imposing water stress. We established two competition treatments by adding nothing (control, CON) or 0.5 g each of *Schizachyrium scoparium* (Michx.) Nash (little bluestem) and *Leersia oryzoides* (L.) Sw. (rice cutgrass) seeds at the time of *Triadica* seed addition (competition, COMP). We chose these species to ensure that natives were present and alive to compete with *Triadica* in both types of water stress. Both have relatively broad moisture tolerances, but *Schizachyrium* tolerates substantial drought whereas *Leersia* tolerates persistent flooding. We established two fertilization treatments by adding water (control, CON) or 4 g/m^2^ nitrogen, 1.3 g/m^2^ phosphorus, 2.7 g/m^2^ potassium and micronutrients (Ultra Turf fertilizer; Vigoro Corp., Illinois, USA) dissolved/suspended in water at seed addition and 8 weeks later (fertilized, FERT). We established treatments for type of water stress by either blocking drainage and “topping off” pots at watering so 8–10 cm of standing water persisted (flood, FLD) or discontinuing watering altogether (drought, DRT) after the designated period of favorable conditions ended. We watered according to treatments and weeded pots not subject to competition thrice weekly. In each pot we quantified *Triadica* abundance, height and leaf count of individual *Triadica* seedlings, and percent cover (visual estimate) and maximum height of native plants at the onset of water stress and 14 and 28 days later. After final surveys we harvested living aboveground biomass of all *Triadica* seedlings from all treatments, native plants from competition treatments, and root biomass of living *Triadica* seedlings from 12-week window treatments. Biomass samples were oven-dried at 70°C for 48 h and weighed.

### Analyses

To evaluate effects of experimental treatments on *Triadica* abundance, we fit abundance (count) data using generalized linear models (GLMs; ‘glm’ in R 2.13; R Foundation for Statistical Computing, Vienna, Austria) with Poisson probability distributions. We used analysis of deviance (ANODEV, a form of likelihood ratio testing; ‘anova’ in R) with chi-squared tests to determine whether window duration, competition, fertilization, stress type and/or their interactions influenced final seedling abundance or the number of germinants or deaths during stress. We analyzed pre-stress seedling abundance the same way but excluded stress type as a model term because stress had not yet been imposed. For abundances of seedlings and germinants we considered all pots (n = 400). For deaths we considered pots that had at least one *Triadica* seedling at at least one survey (n = 111). Importantly, there were zero *Triadica* seedlings in ∼3/4 of the pots. High proportions of zeroes are often suggestive of over-dispersed and zero-inflated data.

To explore this possibility, we fit abundance data using zero-inflated regression models for count data (‘zeroinfl’ in R, package ‘pscl’) and compared models using negative binomial versus Poisson probability distributions. For each response variable, the dispersion parameter (theta) in the negative binomial model was highly insignificant (*p*>0.94), and z-scores and *p*-values for all model terms were highly similar between corresponding models. The same held true if we ignored zero-inflation and compared negative binomial GLMs (‘glm.nb’ in R, package ‘MASS’) with our initial Poisson GLMs. Lastly, we performed likelihood ratio tests for over-dispersion in count data using ‘odTest’ in R (package ‘pscl’). For each response variable we failed to reject the null hypothesis that data are not over-dispersed (initial abundance: p = 0.31, final abundance: p = 0.42, interval germination: p = 0.45). We also found no evidence that zero-inflation (a type of overdispersion) was a factor in our analyses. First, ‘odTest’ should detect zero-inflation but did not. Second, none of the zero-inflation model terms in any of our zero-inflated models were significant. These findings provide strong evidence that our count data are neither over-dispersed nor zero-inflated, and they support our use of Poisson GLMs for abundance data.

We also used logistic regression to directly address whether absence of *Triadica* seedlings depended on treatments and to further investigate the reliability of our Poisson GLMs. We performed logistic regressions by fitting binary *Triadica* presence/absence data using ‘glm’ in R with a binomial distribution and the ‘logit’ link function. We then used ANODEVs with chi-squared tests to evaluate whether treatments influenced pre-stress or final seedling presence or presence of germinants. Results showed that exactly the same set of factors and interactions that significantly influenced seedling abundances also significantly predict presence/absence of *Triadica* seedlings. This further validates the results of our Poisson GLMs. Our zero-inflated and logistic regression analyses considered all pots (n = 400).

This abundance of zeroes did complicate our analyses of seedling performance. Only 92 pots had at least one *Triadica* seedling at the pre-stress survey, and 95 pots did at the final survey. Crucially, the number of pots with seedlings present depended on treatments and resulted in unbalanced sample sizes. Therefore, we performed ANOVAs utilizing Type II Sums of Squares (‘Anova’ in R, package ‘car’) to test whether treatments and/or their interactions influenced pre-stress and final sums of *Triadica* seedling heights and leaf abundances, sums of *Triadica* stem, leaf, root, aboveground and total biomasses, absolute changes (final – initial) in seedling heights and leaf abundances; and root:shoot. Response variables incorporating root biomass include the 12 week window treatment only and were log*_e_* transformed, absolute changes were untransformed, and all other *Triadica* performance metrics were square root transformed for analyses. Due to unbalanced sample sizes, degrees of freedom were sparse and missing values produced unreliable covariance structure among higher order interactions for some response variables. Therefore, we simplified the most complex models by removing all third and fourth order interaction terms. Doing so rectified these issues, resulting in constant degrees of freedom across response variables and consistent output among alternative models. The order of terms can have particularly strong effects on model output with unbalanced designs, thus we used Type II SSs because they do not depend on order of terms. Models using Type III SSs are also suitable and produced equivalent results.

We used Holm-Bonferroni adjusted pairwise t-tests to identify differences between treatment means. We used pot sums instead of averages because we are more interested in population level effects and because averages are confounded by uncontrolled variation in *Triadica* seedling ages.

## Results

### Main Effects

**Figure 1:**
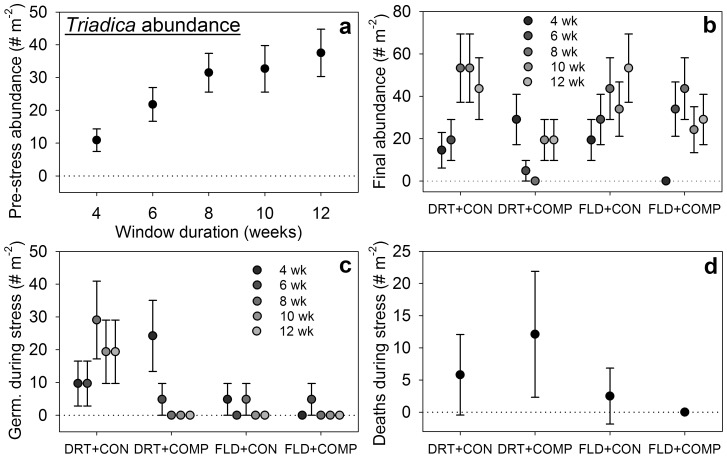
Effects of experimental treatments on four metrics of *Triadica* abundance. Panels (a–d) represent abundance metrics (means ±1 SE) broken down by treatments that significantly affected that metric. Legend: stress type – drought (DRT) or flood (FLD); competition – natives added (COMP) or control (CON). (a) Pre-stress *Triadica* abundance was higher in longer window duration treatments. (b) Final abundance was higher with longer window durations and generally lower with drought stress or competition. (c) Flood or competition generally reduced germination during stress; more germinants were observed after shorter windows but only with competition. (d) More seedlings died during stress when subject to drought or competition.

**Figure 2:**
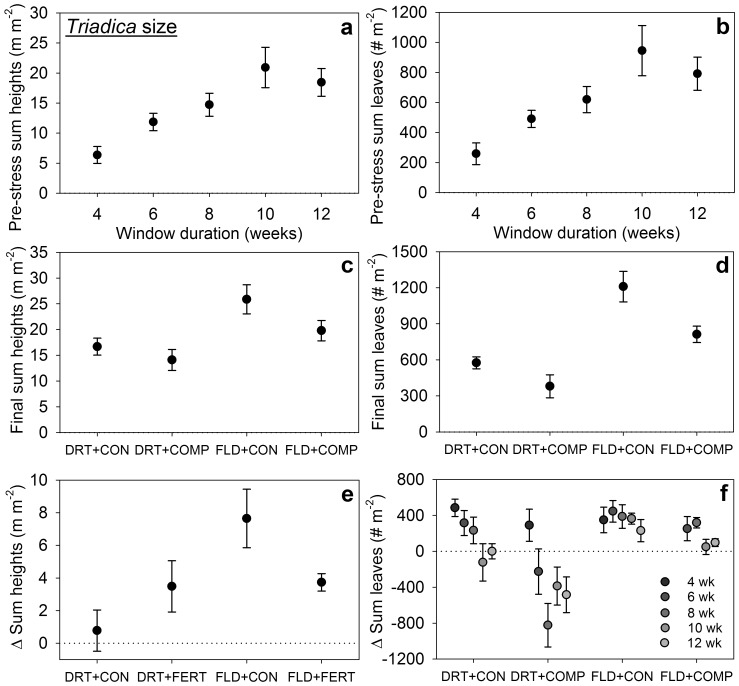
Effects of experimental treatments on **six metrics of** ***Triadica*** **size.** Panels (a–f) represent size metrics (means ±1 SE) broken down by treatments that significantly affected that metric. Legend: stress type – drought (DRT) or flood (FLD); competition – natives added (COMP) or control (CON); fertilization – NPK added (FERT) or control (CON). Pre-stress pot sums of *Triadica* seedling heights (a) and leaf abundances (b) were higher in longer window duration treatments. Final pot sums of seedling heights (c) and leaves (d) were lower with drought or competition. Absolute changes (Δ) during stress in pot sums of *Triadica* seedling heights (e) and leaf abundances (f) were lower and sometimes negative in drought stress and lower with competition. Fertilization increased changes in height in drought but decreased them in flood (e). Changes in leaves decreased with window duration (f), and decreases in changes in leaves with competition were greater in drought (f).

Window duration influenced pre-stress and final *Triadica* seedling abundance (Table 1) and presence (Table S1), pre-stress sums of *Triadica* heights and leaves (Table 2), and changes in leaf abundance during stress (Table 3). All increased as window duration increased (Figs. 1a–b, 2a–b), except changes in leaf abundance which decreased (Fig. 2f). Final *Triadica* abundances in 8 and 12 weeks window treatments were 2.2-fold higher than the 4 weeks treatment (*p* = 0.026 and 0.018, respectively; *p* = 0.051 for 10 versus 4 weeks treatments). Final heights and leaves and stem, leaf and aboveground biomasses were 62%, 24% and 2.4-, 2.0- and 2.2-fold greater, respectively, in longer windows than in the shortest, but these differences were not significant.

**Table 1:** Results of ANODEVs testing effects of experimental treatments on *Triadica* abundance.

		pre-stress abundance			final abundance		germinants during stress			deaths during stress	
Factor	d.f.	?^2^	*p*	d.f.	?^2^	*p*	?^2^	*p*	d.f.	?^2^	*p*
Window	4	15.7	**0.0035**	4	10.4	**0.0349**	2.7	0.61	4	0.5	0.97
Stress				1	1.0	0.31	18.6	**<0.0001**	1	16.2	**<0.0001**
Comp	1	1.5	0.22	1	9.4	**0.0021**	6.5	**0.0106**	1	5.7	**0.0165**
Fert	1	0.7	0.39	1	1.4	0.23	0.3	0.56	1	0.0	0.85
W*S				4	7.2	0.13	2.6	0.63	4	6.0	0.20
W*C	4	0.3	0.99	4	1.6	0.81	14.8	**0.0052**	4	1.2	0.87
W*F	4	1.3	0.87	4	2.5	0.64	4.0	0.41	4	0.6	0.99
S*C				1	2.8	0.09	0.0	0.84	1	2.6	0.09
S*F				1	0.4	0.54	0.4	0.55	1	1.9	0.17
C*F	1	0.9	0.34	1	0.0	0.92	0.1	0.71	1	0.0	0.88
W*S*C				4	17.2	**0.0018**	3.9	0.42			
W*S*F				4	4.9	0.30	2.0	0.73			
W*C*F	4	3.6	0.47	4	2.0	0.73	1.6	0.80			
S*C*F				1	0.0	0.92	0.0	1.00			
W*S*C*F				4	2.7	0.60	0.0	1.00			

Experimental treatments include window duration (W), stress type (S), competition (C), fertilization (F) and their interactions. Pre-stress and final abundances are the numbers of live *Triadica* seedlings observed before and after 28 days of water stress, respectively. Germinants and deaths during stress are abundances of those instances observed during this stress period.

**Table 2:** Results of ANOVAs testing effects of experimental treatments on *Triadica* performance.

		pre-stress sum of heights		pre-stress sum of leaves			final sum of heights		final sum of leaves		stem biomass		leaf biomass		aboveground biomass	
Factor	d.f.	F_91_	*p*	F_91_	*p*	d.f.	F_94_	*p*	F_94_	*p*	F_94_	*p*	F_94_	*p*	F_94_	*p*
Window	4	4.5	**0.0026**	4.8	**0.0017**	4	2.0	0.11	0.2	0.92	1.5	0.21	0.5	0.71	0.8	0.55
Stress	1	2.9	0.09	2.3	0.13	1	14.2	**0.0003**	47.1	**<0.0001**	30.8	**<0.0001**	39.1	**<0.0001**	36.5	**<0.0001**
Comp	1	0.5	0.46	2.8	0.10	1	6.1	**0.0157**	17.3	**<0.0001**	8.7	**0.0043**	11.0	**0.0014**	10.0	**0.0022**
Fert	1	1.7	0.20	0.6	0.46	1	0.5	0.50	0.2	0.69	1.1	0.29	0.5	0.49	0.7	0.40
W*S	4	0.4	0.82	0.5	0.75	4	0.3	0.87	2.2	0.08	1.0	0.42	1.8	0.14	1.3	0.29
W*C	4	0.9	0.49	1.6	0.18	4	1.9	0.11	1.7	0.15	2.3	0.07	3.2	**0.0177**	2.9	**0.0264**
W*F	4	1.1	0.38	1.2	0.32	4	2.3	0.06	1.6	0.19	1.1	0.36	1.3	0.26	1.1	0.35
S*C	1	0.3	0.61	1.2	0.28	1	1.3	0.25	1.3	0.26	3.8	0.06	5.0	**0.0290**	4.8	**0.0314**
S*F	1	0.1	0.73	0.0	0.84	1	0.1	0.73	0.1	0.71	2.7	0.11	3.3	0.07	3.6	0.06
C*F	1	0.9	0.36	2.1	0.16	1	0.1	0.70	0.4	0.52	5.3	**0.0245**	4.0	**0.0489**	5.4	**0.0231**

Experimental treatments include window duration (W), stress type (S), competition (C), fertilization (F) and their interactions. Pre-stress and final sums of heights and leaves are summed totals within individual mesocosm pots of *Triadica* seedling heights and leaf abundances observed before and after 28 days of water stress, respectively. Stem, leaf and aboveground biomasses are sums of dry tissue masses collected from individual pots after 28 days of water stress. All values were square root transformed for analyses.

**Table 3:** Results of ANOVAs testing effects of experimental treatments on absolute changes (Δ) in *Triadica* performance.

		Δ sum of heights		Δ sum of leaves	
factor	d.f.	F_121_	*p*	F_121_	*p*
Window	4	2.4	0.05	4.3	**0.0028**
Stress	1	12.7	**0.0006**	17.9	**<0.0001**
Comp	1	24.2	**<0.0001**	15.4	**0.0002**
Fert	1	0.1	0.71	0.2	0.66
W*S	4	0.4	0.84	0.5	0.75
W*C	4	1.3	0.29	0.3	0.85
W*F	4	0.5	0.75	0.3	0.85
S*C	1	3.9	0.05	4.3	**0.0402**
S*F	1	4.8	**0.0307**	1.6	0.21
C*F	1	1.6	0.21	2.4	0.12

Treatments include window duration (W), stress type (S), competition (C), fertilization (F) and their interactions. Absolute changes (final – initial; Δ) in sums of *Triadica* seedling heights and leaf abundances for each pot were untransformed for analyses. Initial values were measured immediately before we initiated water stress (pre-stress). Final values were measured after 56 days of either drought or flood stress (post-stress). Analyses included pots that had at least one live *Triadica* seedling at the initial or final survey (n = 122).

**Figure 3:**
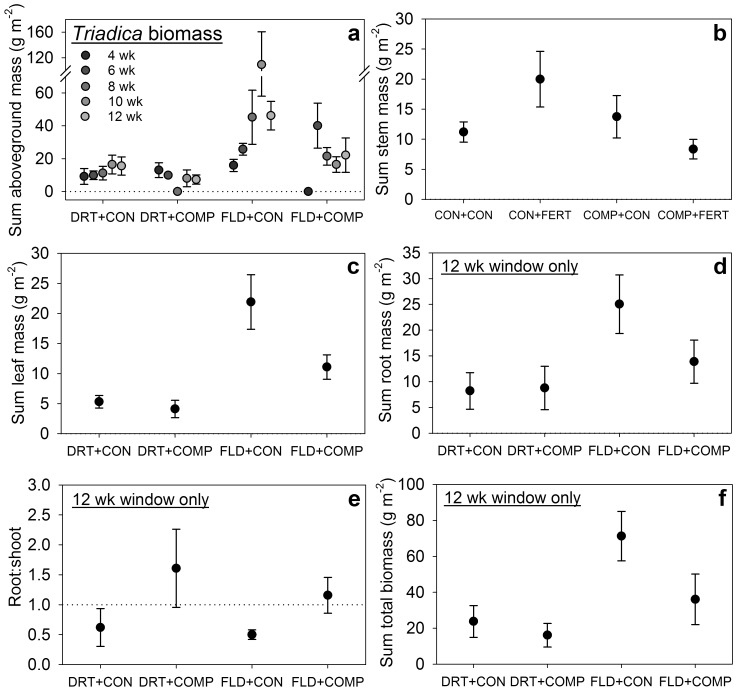
Effects of experimental treatments on six metrics of *Triadica* biomass. Panels (a–f) represent biomass metrics (means ±1 SE) broken down by treatments that significantly affected that metric (except in C where window effects are not show and E-G where insignificant trends are shown). Legend: stress type – drought (DRT) or flood (FLD); competition – natives added (COMP) or control (CON); fertilization – NPK added (FERT) or control (CON). Pot sums of aboveground biomass (a), stem biomass (b) and leaf biomass (c) were reduced in drought or competition treatments, and increased with fertilization without competition but decreased with fertilization with competition (e.g. panel b). Aboveground and leaf biomasses increased with window duration, but these relationships were inconsistent with competition (e.g. panel a). Competition reduced aboveground and leaf biomasses in flood only, but they were lowest in drought overall (e.g. panel c). Sums of root (d) and whole plant biomass (f) were lower in drought treatments. (f) Competition increased root:shoot ratios.

Stress type affected abundances of *Triadica* germinants and deaths during stress (Table 1), presence of germinants during stress (Table S1), final heights and leaves, *Triadica* stem, leaf, root, aboveground and total biomasses (Table 2), and changes in heights and leaves during stress (Table 3). Germinants and deaths were rare during flood stress but ∼10-fold more frequent during drought (Fig. 1c). Otherwise, drought typically reduced *Triadica* performance; flood treatments demonstrated 46% greater final height, 2-fold greater final leaf abundance, 2.6-fold greater changes in height, 10-fold greater changes in leaf abundance (Figs. 2c–f), 2.8-fold more stem biomass, 3.5-fold more leaf biomass, and 3.1-fold more aboveground biomass (Figs. 3a–c). In 12 week window treatments, flood treatments increased *Triadica* root biomass 2.5-fold (F_1,23_ = 5.2, *p = *0.0364) and total biomass 2.8-fold versus drought (F_1,23_ = 6.3, *p = *0.0231; Fig. 3d–f).

Competition with native grasses impacted final *Triadica* seedling abundance and numbers of germinants and deaths during stress (Table 1), final presence of *Triadica* seedlings and presence of germinants (Table S1), final heights and leaves, *Triadica* stem, leaf and aboveground biomasses (Table 2), root:shoot, and changes in heights and leaves (Table 3). Competition decreased final seedling abundance 44% by reducing germinants 60% and increasing deaths 2.8-fold (Fig. 1b–d). Final presence of seedlings and presence of germinants decreased 44% and 68% with competition, respectively. Competition reduced final *Triadica* heights 16%, leaf abundance 26%, and stem, leaf and aboveground biomass by 33–36% (Fig. 2c–d, 3a–c). *Triadica* growth during stress was essentially arrested with competition. Competition reduced increases in heights 99% (approximately zero change) and reduced increases in leaf abundance 116% (leaves were lost; Fig. 2e–f). Among 12 week window treatments, competition increased root:shoot 2.4-fold (F_1,23_ = 7.2, *P* = 0.0165; Fig. 3e).

Fertilization had no effect on abundance or presence of *Triadica* seedlings, germinants or deaths (Tables 1, S1). *Triadica* size and mass were independent of main effects of fertilization (Table 2), but depended on interactions with fertilization discussed below.

### Interactions

The interaction of window duration and competition influenced abundance of *Triadica* germinants during stress (Table 1) and leaf and aboveground biomasses (Table 2). Germinant abundance was generally consistent across window treatments without competition, but it decreased as window duration increased with competition (Fig. 1c). Leaf and aboveground biomasses increased with window duration without competition, but not with competition (Fig. 2a). The 3-way interaction of window, stress type and competition affected final *Triadica* abundance and presence (Tables 1, S1). Without competition, abundances were greater in longer window treatments regardless of stress type. With competition, abundances in drought pots were highest in the shortest window and lowest in intermediate windows, whereas the opposite was true in flood pots (lowest in short, highest in intermediate; Fig. 1b). Final *Triadica* presence followed the same general pattern.

The interaction of stress type and competition affected *Triadica* leaf and aboveground biomasses and changes in leaf abundance (Tables 2, 3). In drought treatments, these biomasses were lowest and competition insignificantly reduced both. In flood treatments, biomasses were >3-fold higher overall and competition significantly reduced them 46–50% (Fig. 3a,c). Changes in leaf abundance were insignificantly lower with competition in flood, but significantly lower and negative with competition in drought (Fig. 2f). Changes in sums of heights depended on the interaction of stress and fertilization (Table 3). Fertilization increased vertical growth 4.5-fold during drought, but reduced increases 51% during flooding. Increases in height were similar in drought and flood treatments with fertilization.

The interaction of competition and fertilization influenced final *Triadica* stem, leaf and aboveground biomasses (Table 2). Fertilization increased these 79%, 57% and 69%, respectively, without competition but reduced them 39%, 35% and 37%, respectively, with competition.

Experimental treatments also influenced performance of native plants. Analyses and results pertaining to native plants are presented in the supporting information (Appendix S1, Fig. S1, Table S2).

## Discussion

Reinvasion pressure is vital to restoration outcomes and costs, but it can vary widely among equivalently invaded habitats and is difficult to predict [1]–[3]. Invasive *Triadica sebifera* (Chinese tallow tree) exhibits rapid ontogenetic niche expansions in its moisture tolerance early in life [8]. This likely contributes to broad variation in average reinvasion pressure among restorations of *Triadica*-dominated ecosystems [7], [8]. For plants with expanding abiotic niches like *Triadica*, our ‘outgrow the stress’ hypothesis holds that reinvasion pressure is determined by spatiotemporal availability of realized recruitment windows when exotic propagules are abundant [2]. This study tested two basic tenants of this hypothesis. (1) There is a minimum establishment time for exotics wherein they must germinate and grow to a stage and/or size capable of tolerating subsequent conditions. (2) Factors influencing growth can influence individual attainment of required tolerances and thus permit or preclude recruitment. Our results were not this black and white, but they clearly validate the core predictions of the ‘outgrow the stress’ hypothesis below.

### Prediction 1

Recruitment success will scale with temporal availability of abiotic windows. Our results clearly show that longer periods of favorable conditions prior to water stress (i.e. greater abiotic window availability) increased *Triadica* abundance before and after subsequent stress periods. Overall, longer windows also increased final *Triadica* size, but benefits were less straightforward because longer windows had insignificant or negative impacts on some response variables in specific environmental contexts. This increased variance across window treatments and resulted in window duration having few significant main effects on size. Few studies have considered ontogenetic niche expansions. To our knowledge there are no other direct experimental tests of whether longer abiotic windows increase recruitment in stressful environments. However, observational studies often link longer periods between stressful events to increased recruitment. For example, Stokes [41] observed enhanced *Salix nigra* recruitment in areas subject to less frequent inundation. Manipulation of abiotic window durations in the field poses significant logistical challenges, but is necessary to experimentally test this prediction in a more natural setting. Window frequency is another aspect of window availability that should affect reinvasion in different ways and merits investigation.

Two metrics of *Triadica* success were reduced among longer window treatments, but only in specific contexts. There were fewer germinants during stress in longer windows with competition (Table 1, Fig. 1c), and seedlings gained fewer or lost more leaves among longer windows during drought (Table 2, Fig. 2f). Window duration also had insignificant or inconsistent effects on leaf and aboveground biomass in drought or competition treatments, respectively (Table 2, Fig. 3a). We expect the mechanism here reflects resource limitation and varying levels of demand. Water was most limiting in drought, and adding natives produced greater demand and competition for water. Longer windows exacerbated water limitation by increasing pre-stress size and abundance of *Triadica* seedlings and native plants (Figs. 1a, 2a–b, S1a–b), which produced higher total water demand. We observed similarly decreased success among the largest seedlings in the driest moisture treatments when investigating *Triadica*’s moisture niche expansions [8]. Reduced *Triadica* growth during flood stress with competition likely reflects reduced light availability (Figs. 2c–d, 3a–c). Fertilization may augment this effect during flood (Figs. 2e, 3b). Notably, we previously observed peak flood-induced mortality among *Triadica* seedlings shorter than sustained flood depths [8]. This likely explains mortality and final seedling abundances of zero observed in 4 week window treatments with flooding, which may reflect a strict minimum establishment time.

### Prediction 2

If size confers tolerance [32], factors that increase or decrease growth rates will have similar effects on recruitment of plants with expanding niches during finite windows of opportunity in stressful environments. Our results clearly show that competition with native plants decreased *Triadica* size and mass, as well as abundance. Fertilization increased *Triadica* performance in some contexts, but did not affect abundance. This may be because field soils utilized were relatively fertile. Total N content in soils near our collection site was 0.26% of dry mass in 2009 (Gabler and Siemann, unpublished data). Stress type treatments produced differences in size and mass often larger than between competition treatments, and it had strong effects on germinant and death abundances. Yet, stress type only affected final seedling abundance in interaction with window duration and competition. Decreases in *Triadica* abundance in drought treatments are unlikely to have been an effect of enhanced performance of the drought-tolerant native because native performance also decreased in drought (Appendix S1, Figure S1, Table S2). Rather, what were likely relatively weaker native competitors had a stronger overall competitive effect, presumably due to the scarcity of water.

Our findings largely support this prediction of the ‘outgrow the stress’ hypothesis. It holds that changes in growth rate affect recruitment by altering minimum establishment times, but it acknowledges that differences in required establishment time do not mandate differences in recruitment. Recruitment ultimately depends on whether minimum establishment times exceed the duration of favorable conditions available locally. Environmental factors should only influence recruit abundance when factors shorten or extend required establishment times across the critical threshold of local duration of suitable conditions. Size and mass are meaningful aspects of recruitment success and reinvasion pressure in their own rights, and may affect future survival in manifold ways.

### Implications for Restoration and Management

We quantified baseline establishment times for *Triadica* across a variety of realistic environmental scenarios that are consistent with its documented ontogenetic niche expansions [8] and observed restoration outcomes in previously *Triadca*-dominated habitats [7] (Gabler and Siemann, unpublished data). We quantified how key factors interact to influence *Triadica* establishment and growth. These results increase our understanding of the mechanisms of reinvasion in *Triadica* and generally, and may improve predictions of exotic recruitment based on local conditions [2], [31]. Enhanced understanding and predictions can inform management of *Triadica* and other exotics exhibiting niche expansions.

Our findings concern some phenomena beyond our control (e.g. drought). However, managers can take advantage of these events to increase management efficacy or efficiency. Native addition is commonly recommended to suppress invaders [3], [42]. However, the literature disagrees over whether this is effective, especially for superior competitors like *Triadica* [3]. Adding natives decreased *Triadica* establishment and growth in this study, but not in all contexts. Results suggest fertilization can reduce *Triadica* performance in some contexts, but we do not recommend this because fertilization can have negative effects. The reinvasion pressure framework validated here can inform site selection and exotic management strategies during restoration or control efforts, especially where exotics exhibit ontogenetic niche expansions.

## Supporting Information

Figure S1 **Effects of experimental treatments on native plants.**
(PDF)

Table S1 **Results of ANODEVs using logistic regression models to test effects of experimental treatments on** ***Triadica*** **presence.**
(PDF)

Table S2 **Results of ANOVAs testing effects of experimental treatments on native plant abundance and performance.**
(PDF)

Table S3 **Experimental data used in this study.**
(CSV)

Appendix S1 **Analyses and results of experimental treatments on native plants.**
(PDF)
