# Supplementary material for: Timing of Favorable Conditions, Competition and Fertility Interact to Govern Recruitment of Invasive Chinese Tallow Tree in Stressful Environments
Source: PLoS One. 2013 Aug 13;8(8):e71446. doi: 10.1371/journal.pone.0071446 (PMC3742752; doi:10.1371/journal.pone.0071446)
Supplement: Figure S1 — Effects of experimental treatments on native plants. (PDF) [file pone.0071446.s001.pdf]

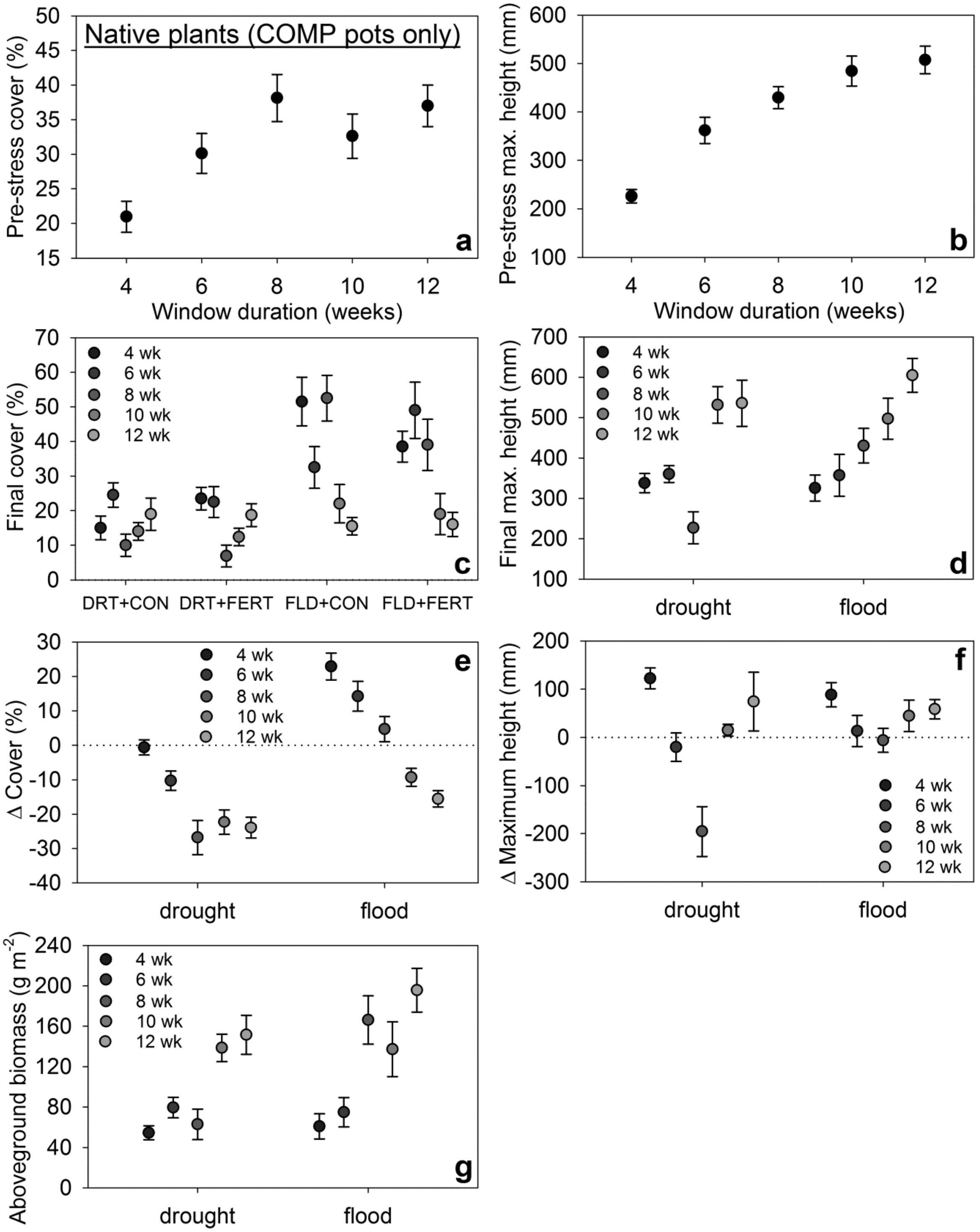

Figure S1. Effects of experimental treatments on native plants.
Panels represent metrics of native plant performance (means ±1 SE) broken down by treatments that significantly affected that metric. Legend: stress type – drought (DRT) or flood (FLD); fertilization – NPK added (FERT) or control (CON). Only pots with the competition treatment are considered because native plants were weeded in non-competition treatments. Pre-stress

native plant percent cover (a) and maximum height (b) were greater among longer window duration treatments. (c) Final percent cover was lower in drought stress and decreased with window duration in flood treatments. Fertilization influenced final cover in some window treatments but followed no clear pattern. (d) Final maximum height was greater among longer window treatments, and was greater in flood stress in the 8 week window duration treatment only. (e) Absolute changes in native plant generally decreased with window duration and were lower and typically negative in drought stress. Decreases in native cover were consistent among longer window treatments during drought only. (f) Changes in height were lowest and sometimes negative among intermediate window durations. Decreases among intermediate windows were exaggerated during drought. (g) Native plant aboveground biomass generally increased with window duration, and was greater in flood stress in 8 and 12 week window duration treatments only.
