# Supplementary material for: Timing of Favorable Conditions, Competition and Fertility Interact to Govern Recruitment of Invasive Chinese Tallow Tree in Stressful Environments
Source: PLoS One. 2013 Aug 13;8(8):e71446. doi: 10.1371/journal.pone.0071446 (PMC3742752; doi:10.1371/journal.pone.0071446)
Supplement: Table S1 — Results of ANODEVs using logistic regression models to test effects of experimental treatments on Triadica presence. (PDF) [file pone.0071446.s002.pdf]

Table S1. Results of ANODEVs using logistic regression models to test effects of experimental treatments on *Triadica* presence.

		pre-stress presence			final presence		germination during stress	
**Factor**	**d.f.**	χ^2	***p***	**d.f.**	χ^2	***p***	χ^2	***p***
Window	4	11.4	**0.0225**	4	9.7	**0.0460**	3.0	0.56
Stress				1	0.4	0.55	17.4	**<0.0001**
Comp	1	1.5	0.23	1	10.4	**0.0012**	7.9	**0.0048**
Fert	1	0.5	0.47	1	1.8	0.18	0.0	0.83
W*S				4	6.9	0.14	2.3	0.67
W*C	4	0.7	0.95	4	1.2	0.87	13.4	**0.0094**
W*F	4	0.9	0.92	4	0.6	0.96	3.2	0.53
S*C				1	2.4	0.12	0.0	0.90
S*F				1	0.8	0.37	0.3	0.58
C*F	1	0.2	0.67	1	0.0	1.00	0.3	0.61
W*S*C				4	14.5	**0.0059**	3.8	0.43
W*S*F				4	3.1	0.54	2.1	0.72
W*C*F	4	5.2	0.27	4	2.6	0.63	1.6	0.82
S*C*F				1	0.1	0.76	0.0	1.00
W*S*C*F				4	2.7	0.60	0.0	1.00

Experimental treatments include window duration (W), stress type (S), competition (C), fertilization (F) and their interactions. Pre-stress and final presence denote whether any live *Triadica* seedlings (>0) were observed before and after 28 days of water stress, respectively. Germinantion during stress denotes whether we observed any newly germinated seedlings during this stress period.
