# Supplementary material for: Timing of Favorable Conditions, Competition and Fertility Interact to Govern Recruitment of Invasive Chinese Tallow Tree in Stressful Environments
Source: PLoS One. 2013 Aug 13;8(8):e71446. doi: 10.1371/journal.pone.0071446 (PMC3742752; doi:10.1371/journal.pone.0071446)
Supplement: Table S2 — Results of ANOVAs testing effects of experimental treatments on native plant abundance and performance. (PDF) [file pone.0071446.s003.pdf]

Table S2. Results of ANOVAs testing effects of experimental treatments on native plant abundance and performance.

		pre-stress percent cover		final percent cover		pre-stress max height		final max height		aboveground biomass		Δ percent cover		Δ max height	
**factor**	**d.f.**	F_{199}	*p*	F_{199}	*p*	F_{199}	*p*	F_{199}	*p*	F_{199}	*p*	F_{199}	*p*	F_{197}	*p*
Window	4	5.1	**0.0006**	9.7	**<0.0001**	19.5	**<0.0001**	14.6	**<0.0001**	10.2	**<0.0001**	27.1	**<0.0001**	11.2	**<0.0001**
Stress	1	1.7	0.20	60.9	**<0.0001**	0.0	0.92	2.8	0.09	4.5	**0.0351**	83.3	**<0.0001**	3.8	0.05
Fert	1	0.0	0.90	0.3	0.60	1.7	0.19	0.4	0.55	2.6	0.11	0.5	0.47	0.4	0.52
W*S	4	1.5	0.21	10.3	**<0.0001**	1.1	0.34	2.7	**0.0336**	4.0	**0.0039**	3.6	**0.0081**	3.5	**0.0083**
W*F	4	0.5	0.74	1.5	0.20	0.1	0.97	1.4	0.24	1.6	0.18	0.7	0.60	1.5	0.21
S*F	1	1.2	0.28	0.4	0.51	0.1	0.75	0.5	0.50	0.0	0.93	0.6	0.45	0.2	0.65
W*S*F	4	0.6	0.65	2.6	**0.0397**	1.1	0.38	1.7	0.16	1.1	0.38	0.5	0.72	1.3	0.28

Treatments include window duration (W), stress type (S), fertilization (F) and their interactions. These tests only consider pots subject to the competition treatment because native plants were weeded from non-competition pots; hence competition is not considered as a factor here. Pre-stress and final native plant percent cover were arcsine transformed, pre-stress and final maximum native plant height were untransformed, aboveground native plant biomass was square root transformed, and absolute changes (Δ) in cover and maximum height during stress were untransformed for analyses.
