# Supplementary material for: Timing of Favorable Conditions, Competition and Fertility Interact to Govern Recruitment of Invasive Chinese Tallow Tree in Stressful Environments
Source: PLoS One. 2013 Aug 13;8(8):e71446. doi: 10.1371/journal.pone.0071446 (PMC3742752; doi:10.1371/journal.pone.0071446)
Supplement: Appendix S1 — Analyses and results of experimental treatments on native plants. (PDF) [file pone.0071446.s005.pdf]

Appendix S1. Analyses and results of experimental treatments on native plants.

For pots with native competition, we fit GLMs and used ANOVAs to evaluate treatment effects on native plants in terms of their pre-stress and final percent cover (arcsine transformed) and maximum height (untransformed), final aboveground biomass (square root transformed) and absolute changes in cover and maximum height (untransformed). We used Holm-Bonferroni adjusted pairwise t-tests to identify differences between treatment means. We used pot sums instead of averages because we are more interested in population level effects and because averages are confounded by uncontrolled variation in *Triadica* seedling ages.

Window duration influenced all native plant response variables: pre-stress and final percent covers and maximum heights, final aboveground biomass, and changes in percent cover and maximum height during stress (Table S2). Pre-stress native cover, pre-stress and final native heights and biomass increased with window duration (Fig. S1a,b,d,g). Final cover and changes in cover decreased as window duration increased (Fig. S1c,e). Changes in height were lowest in intermediate windows durations (Fig. S1f). Stress type also influenced final native plant cover, biomass and change in cover (Table S2). Native plants exhibited 2-fold more final ground cover and 30% more biomass in flood treatments versus drought. Native cover increased 3% during stress in flood but decreased 17% in drought. Native plants were only present in competition pots, so the competition treatment does not apply to native plant response variables. Fertilization had no main effects on native plants.

The interaction of window duration and stress type influenced final percent cover and maximum height, biomass, and changes in cover and maximum height (Table S2). In flood treatments only, final cover was lower in 10 and 12 week treatments than in shorter windows. Final height and biomass increased with window in flood treatments, but were reduced in intermediate windows in drought treatments. Decreases in change in height among intermediate windows were exaggerated with drought. Changes in cover decreased faster as window increased and were always negative in drought treatments. Final percent cover also depended on the interaction of window, stress and fertilization (Table S2). Increases in cover typically associated with flood or fertilization treatments were not observed in the 10 and 12 week window treatments (Fig. S1c).
